# Determinants for the implementation of a combined lifestyle intervention for patients with knee osteoarthritis and overweight: a qualitative study

**DOI:** 10.1136/bmjopen-2025-108216

**Published:** 2026-01-28

**Authors:** Priya Gharbaran, Nuria EJ Jansen, Inge Merkelbach, Marienke van Middelkoop, Dieuwke Schiphof

**Affiliations:** 1 General Practice, Erasmus MC University Medical Center Rotterdam, Rotterdam, The Netherlands; 2 Erasmus School of Social and Behavioural Sciences / Behavioural Change, Erasmus Universiteit Rotterdam, Rotterdam, The Netherlands

**Keywords:** Behaviour, Exercise, Obesity, Primary Care, QUALITATIVE RESEARCH

## Abstract

**Abstract:**

**Objectives:**

Lifestyle changes—such as adopting healthy nutrition and increasing physical activity—are essential for alleviating symptoms in patients with knee osteoarthritis (OA) and overweight, with weight loss being a key outcome of these changes. Since 2019, healthcare professionals (HCPs) in the Netherlands have been able to refer these patients to a reimbursed combined lifestyle intervention (CLI). This study aims to identify determinants affecting CLI implementation for individuals with knee OA and overweight from both patient and HCP perspectives.

**Design:**

Semistructured interviews were conducted in a qualitative study with 23 individuals with knee OA and overweight and 16 HCPs (general practitioners (GPs) and lifestyle coaches). Interviews were transcribed verbatim and coded independently by two researchers using the updated Consolidated Framework for Implementation Research (CFIR).

**Setting:**

Primary care, including GPs and lifestyle coaches from the Greater Rotterdam region in the Netherlands.

**Participants:**

23 individuals with knee OA and overweight and 16 HCPs (GPs and lifestyle coaches).

**Results:**

Determinants were explored within four CFIR domains: innovation, outer setting, inner setting and individuals. Key facilitators included recognition of the programme’s potential, strong social support and positive participant–coach relationships. Major barriers involved the absence of an exercise component, financial constraints limiting its inclusion, scepticism among GPs about care quality, limited expertise of lifestyle coaches addressing OA-specific needs and difficulties adapting the programme to participants’ diverse knowledge levels and health literacy.

**Conclusions:**

To improve the implementation of the CLI for patients with knee OA, it is essential to incorporate a tailored exercise component, strengthen lifestyle coaches’ expertise, address financial barriers and build trust among GPs through education and clear communication of programme outcomes. Tailoring the CLI to better meet participant needs is crucial to ensure its long-term effectiveness and sustainability as a treatment for individuals with knee OA and overweight.

**Trial registeration number:**

Netherlands Trial Registry (NL9355).

**STRENGTHS AND LIMITATIONS OF THIS STUDY**

- By including individuals with knee osteoarthritis (OA) and healthcare professionals from various combined lifestyle intervention programmes outside the Lifestyle Intervention Trial (LITE study) and using purposive sampling, we ensured a diverse sample in terms of age, sex, level of education (as proxy for socioeconomic position), health literacy, cultural background and session attendance.
- Since the study was conducted in the Netherlands, the findings may be less generalisable to other countries with different healthcare systems.
- As most individuals with knee OA were recruited through the LITE study, their motivation may differ from patients in standard care.

## Introduction

 Knee osteoarthritis (OA) is a significant global health concern and a leading cause of disability, affecting millions worldwide.^1^ As an age-related condition, its prevalence is increasing in response to the growing proportion of older individuals in the global population. In the Netherlands, an estimated 1.6 million individuals are affected, though primary care data suggest the true prevalence could be nearly twice as high.^2^ By 2040, OA is projected to become the most common chronic disease in the Netherlands.^3^ This trend is further driven by rising rates of overweight, a major risk factor for OA. With over half of Dutch adults classified as overweight in 2023, there is an urgent need for public health interventions.^4 5^

Overweight accelerates the progression of knee OA and worsens symptoms, such as pain, joint stiffness and reduced mobility.^6 7^ This can trigger a vicious cycle of inactivity, muscle weakening and further weight gain, ultimately reducing quality of life and hindering healthy ageing.^8^ Substantial evidence shows that weight loss through exercise therapy and dietary changes is the most effective non-pharmacological approach for managing knee OA symptoms and improving quality of life.^9 10^ While each strategy is moderately effective on its own, their combination provides the greatest benefits by promoting weight loss, which in turn reduces pain and enhances physical function.^11 12^ Given the critical role of weight management in knee OA, many international guidelines recommend 5%–10% weight loss for patients with knee OA and overweight to prevent further clinical and structural progression.^9^

Since 2019, healthcare professionals (HCPs) in the Netherlands have been able to refer patients with obesity or patients with overweight and specific comorbidities, such as OA, to a reimbursed combined lifestyle intervention (CLI) with a duration of 2 years. The general aim of the CLI is at least 5% loss of body weight by promoting physical activity, healthy nutrition and behavioural changes. However, patients with knee OA face unique challenges compared with other CLI target groups, such as individuals with diabetes or cardiovascular disease, due to OA-related pain and fear of movement, which severely limit physical activity.^13^ Given the CLI’s strong emphasis on physical activity, the facilitators and barriers for patients with knee OA are likely to differ from those for other conditions. Barriers such as limited time for motivational interviewing, perceived lack of effectiveness, patient adherence and costs have been well documented in other patient populations.^14 15^ While some barriers may have been mitigated through measures like insurance coverage, additional challenges may still persist.

For successful implementation, lifestyle programmes must align with participant needs and fit within primary care routines. Understanding the determinants that influence implementation—including socioeconomic, cultural and health literacy factors—is essential to enhance the CLI’s reach, acceptance and effectiveness. Therefore, this study aims to identify determinants affecting CLI implementation for individuals with knee OA and overweight from both patient and HCP perspectives.

## Methods

This study is an expansion of the Lifestyle Intervention Trial (LITE study), a randomised controlled trial (RCT) investigating the (cost-)effectiveness of a CLI for patients with early knee OA and overweight or obesity in primary care.^16^ The study population includes individuals diagnosed with knee OA according to the National Institute for Health and Care Excellence (NICE) guideline, with a Body Mass Index (BMI) ≥25, who consulted their general practitioner (GP) for knee complaints within the past 24 months.^17^ Eligible participants were aged between 45 and 70 years.

The LITE study focuses on *BeweegKuur*, one of the seven officially recognised CLI programmes in the Netherlands. While all programmes share the same goal, they vary in structure and content. An overview of the CLI programmes is provided in online supplemental file table S1.

### Study design and conceptual framework

A qualitative design based on the research paradigm of interpretivism was used to explore individual perspectives, emphasising the importance of context in understanding behaviour. Semistructured interviews allowed people with knee OA and HCPs to share their experiences with engaging in or delivering the CLI for knee OA. The updated Consolidated Framework for Implementation Research (CFIR) was used to give direction to the interview questions, coding and data analysis.^18^ The CFIR is a well-established and widely recognised framework in the field of implementation science to assess potential determinants that influence implementation in diverse contexts. The updated CFIR incorporates 48 constructs across five domains: (1) innovation, (2) outer setting, (3) inner setting, (4) individuals and (5) implementation process. In this study, we used the first four domains. Data are reported following the Standards for Reporting Qualitative Research criteria.^19^

The Medical Ethics Review Board of Erasmus MC University Medical Center Rotterdam has assessed the study and concluded that this study did not fall under the Dutch Medical Research Involving Human Subjects Act (Wet Medisch-wetenschappelijk Onderzoek (WMO)) (MEC-2023–0756).

### Study sample

Participants were required to be currently enrolled in or have previously participated in a CLI programme and have knee OA; no other exclusion criteria were applied. Participants from the intervention arm of the LITE study were invited to share their experiences with the CLI in an interview. Purposive sampling was used to ensure diverse representation in terms of age, sex, level of education, health literacy, cultural background and session attendance (eg, complete attendance or early programme disengagement). Additionally, individuals with knee OA enrolled in CLI programmes other than BeweegKuur were invited to provide broader insights into different programme structures and content. These participants, who were not part of the LITE trial, were recruited via the research team’s network and LinkedIn.

HCPs, including GPs and accredited lifestyle coaches (either dietitians or exercise professionals with certified lifestyle coaching training), who were involved in the LITE study, were invited to share their experiences with referring patients or delivering the CLI to individuals with knee OA. Additional HCPs were recruited through the research team’s network, LinkedIn and direct invitations to professionals working in the Rotterdam region.

### Data collection

The semistructured interviews were conducted between December 2023 and May 2024 by two researchers (PG and NEJJ). PG has a background in health sciences, and NEJJ has a background in human movement sciences. Both researchers received training in qualitative interviewing prior to the study. Individuals with knee OA and HCPs gave their written informed consent prior to the start of the interviews. The first two interviews were conducted with two researchers together to ensure methodological consistency, while the remaining interviews were done individually. Three tailored semistructured interview guides were designed, based on the CFIR domains and existing literature, for individuals with knee OA, GPs and lifestyle coaches. These included questions based on the CFIR domains, along with additional topics relevant to the study and informed by existing literature. The initial version was pilot tested with a physiotherapist on the research team to assess clarity and relevance. Minor revisions were made to the guide after the first interview, primarily to refine the wording of questions. The complete interview guides are available in Supplementary File: interview guides. When participants struggled to generate their own examples, prompts were provided to assist them. Interviews lasted 45–60 min and were conducted via Microsoft Teams, where they were video recorded. When online participation was not possible, in-person interviews were held at the Erasmus MC University Medical Center, and these were audio recorded. All interviews were transcribed verbatim. No repeat interviews were conducted, and none of the participants wanted to check the transcripts. Data saturation was considered reached when no new topics emerged during the interviews. This approach was used to ensure that the data adequately captured a representative range of participant perspectives.

Demographic characteristics of individuals with knee OA were collected through a questionnaire that gathered information on age, sex, cultural background, education level and health literacy. Health literacy was assessed using the brief Health Literacy Screening Tool.^20^

Demographic characteristics of HCPs—such as age, sex, cultural background, profession and work setting—were collected at the start of the interview.

### Data analysis

Thematic analysis was used, combining an inductive and a deductive approach, using Max Qualitative Data Analysis (MAXQDA) 2018 (VERBI Software, 2018).^21^ First, two researchers (PG and NEJJ) independently coded two transcripts using an inductive approach to develop initial codes. These codes were then organised into two separate coding trees—one for people with knee OA and one for HCPs—to account for the different perspectives and experiences between the two groups. The coding trees were subsequently refined based on discussion with a third researcher (DS) and insights from the initial transcripts, after which a third transcript was coded using the revised coding tree. After reaching 75% inter-rater reliability of the coding trees, one researcher (PG) coded all remaining transcripts.

Related codes were grouped to form preliminary themes, which were discussed by multiple members of the research team (PG, NEJJ, MvM and DS) to resolve discrepancies. The themes were subsequently mapped onto the constructs of the four CFIR domains used in this study. Descriptive summaries were created for each theme to draw conclusions. The interviews and data analysis were conducted in Dutch, after which results and quotes were translated into English.

To ensure the rigour of the findings, several strategies were used. Credibility was enhanced through data triangulation across participant groups. Dependability was ensured using a structured coding process, including inter-rater reliability checks and team discussions to resolve discrepancies. Confirmability was promoted by mapping the final themes onto the constructs of the CFIR framework, supporting transparency in the analysis process.

### Patient and public involvement

Participants were involved in research through Artrose Gezond (Healthy with Osteoarthritis), a platform for people with OA, coordinated from the Erasmus MC University Medical Center Rotterdam, the Netherlands. The interview question guides were informed by experiences and reflections shared by participants and HCPs. Patients were involved in the design of the study and the data interpretation through two workshops in which the findings were presented and discussed. Additionally, the results were presented in two separate sessions with lifestyle coaches and GPs to gather their perspectives on the findings and to explore how these insights could inform clinical practice.

## Results

A total of 39 individuals were interviewed, including 23 individuals with knee OA and overweight (BMI >25) and 16 HCPs. In addition, several other individuals who were approached for participation declined due to other health conditions (n=3) or time constraints (n=7). Participant characteristics are presented in table 1. For detailed information about the participants, see online supplemental file table S2. The median age of individuals with knee OA was 56 years (range: 45–71), and the majority were female (69.6%). Most participants were able to understand and use health-related information effectively, leading to adequate health literacy scores. However, two participants reported difficulties with reading and comprehending medical information, resulting in marginal health literacy scores.

**Table 1:** Characteristics of study sample (n=39)

Participants (n=23)	
	n (%)
Sex	
Male	7 (30.4)
Female	16 (69.6)
Age	
45–55	10 (43.5)
56–65	10 (43.5)
66–75	3 (13)
Level of education	
Low	7 (30.4)
Middle	9 (39.1)
High	7 (30.4)
Ethnicity	
Dutch	14 (60.9)
Surinamese	4 (17.4)
Mixed Dutch/other	5 (21.7)
Health literacy*	
Adequate	17 (73.9)
Marginal	2 (8.7)
Inadequate	0 (0.0)
Missing	4 (17.4)

Healthcare professionals (n=16)	
	n (%)
Sex	
Male	4 (25.0)
Female	12 (75.0)
Age	
30–40	6 (37.5)
41–50	2 (12.5)
51–60	3 (18.8)
Missing	5 (31.2)
Ethnicity	
Dutch	14 (87.5)
Non-western (not specified)	1 (6.2)
Mixed Dutch/other	1 (6.2)
Occupation	
General practitioner	7 (43.8)
Lifestyle coach (dietitian)	8 (50.0)
Lifestyle coach (exercise professional)†	1 (6.2)

* Health literacy assessed using Brief Health Literacy Screening Tool (BRIEF): inadequate health literacy (3–8), marginal health literacy (9–11) and adequate health literacy (12–15).

† A qualified personal trainer.

The HCPs had a median age of 39 years (range: 31–59) and were predominantly female (75%). Among the 16 HCPs were seven GPs and nine lifestyle coaches. Most lifestyle coaches were dietitians delivering either the BeweegKuur (n=6) or Samen Sportief in Beweging (SSiB) (n=1) programmes. One lifestyle coach, delivering the (Coaching op Leefstijl) CooL programme (n=1), had an academic background. The exercise professional was a qualified personal trainer.

**Figure 1:**
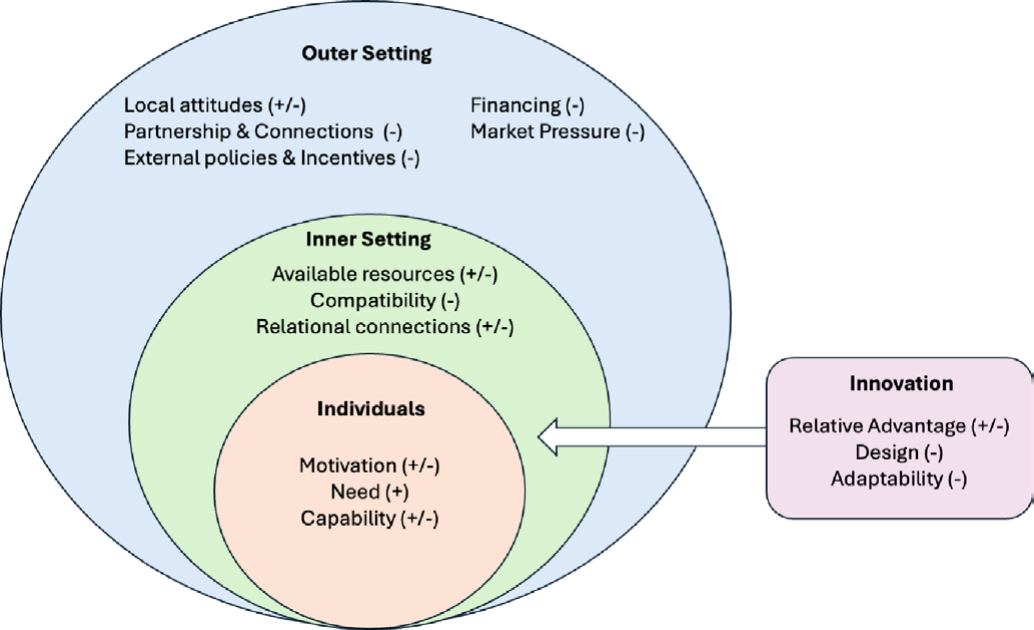
Identified CFIR constructs and their impact on the implementation of the CLI for individuals with knee OA and overweight. ‘−’ indicated barriers; ‘+’ indicated facilitators; CFIR, Consolidated Framework for Implementation Research; CLI, Combined Lifestyle Intervention; OA, osteoarthritis.

Of the 48 CFIR constructs, 14 across four domains were identified as relevant to the implementation of the CLI for individuals with knee OA and overweight (figure 1). Table 2 provides definitions of the identified construct, while online supplemental table S3 summarises all determinants, categorised by CFIR domains for each stakeholder group. Below, we highlight the most important determinants, with ‘Q’ referencing corresponding quotes in online supplemental table S3: table S3: summary of findings by the CFIR domains, constructs and corresponding themes.

**Table 2:** CFIR domains, constructs and definitions for the implementation of the CLI for patients with knee OA

CFIR Domain	CFIR Construct	Definition of construct	Corresponding theme
Innovation domain	Relative advantage	The perceived benefits or superiority of the CLI (compared with current practices).	Perceived effectiveness of the CLI for OA management
	Design	The way the CLI is structured, bundled and presented.	Insufficient physical activity support and programme limitations in the CLI for individuals with knee OA
	Adaptability	The extent to which the CLI can be personalised or adjusted to fit the specific needs or contexts.	Challenges in participation due to diverse knowledge levels and health literacy
Outer setting domain	Local attitudes	The influence of sociocultural values and beliefs on the implementation process.	Sociocultural factors and social support
	External Policies & incentives	The role of external regulations, laws and incentives that either support or hinder implementation.	
	Financing	The availability and sustainability of funding needed for successful implementation.	
	Market Pressure	The external forces, including competition and industry demands, that impact the adoption of the CLI.	Scepticism among GPs regarding the implementation of the CLI
	Partnerships & connections	The collaboration and relationships between stakeholders, organisations and other partners that support or influence the implementation process.	
Inner Setting domain	Available resources	The financial, human and material resources necessary for effective implementation.	
	Compatibility	The alignment of the CLI with existing values, practices and needs of individuals or organisations.	
	Relational Connections	The quality of communication and relationships among individuals involved in the implementation.	Fit to coach
Individuals domain	Need	The perceived necessity of implementing the CLI.	
	Motivation	The willingness and commitment of individuals to engage in and support the implementation of the CLI.	Participant motivation and engagement in lifestyle change
	Capability	The skills and knowledge available to implement the CLI effectively.	Expertise among lifestyle coaches for OA support

CFIR, Consolidated Framework for Implementation Research; CLI, combined lifestyle intervention; GPs, general practitioners; OA, osteoarthritis.

### Domain 1: innovation

The first CFIR domain covers characteristics of the innovation—specifically the CLI—that influence its successful implementation. Key themes include relative advantage (perceived effectiveness for OA management), design (insufficient physical activity support, programme duration) and adaptability (diverse knowledge levels and health literacy).

#### Perceived effectiveness of the CLI for OA management

Nine individuals with knee OA reported making lifestyle changes as a result of the CLI, with seven people reporting weight loss and five noting reduced knee pain after completing the programme (*Q1, CLI participant 9*). These positive outcomes were echoed by five lifestyle coaches, stating that they observed improvements in knee complaints as individuals with knee OA increased their physical activity or lost weight (*Q2, exercise professional 1*). Similarly, all GPs acknowledged that a healthy diet, weight loss and muscle strengthening could alleviate symptoms of OA, with one GP highlighting the CLI’s potential to delay or prevent joint replacement surgeries (*Q3, GP4*).

However, despite these reported benefits, the majority of the people with knee OA (n=16) stated that the CLI did not result in weight loss (*Q4, CLI participant 1*). Furthermore, two GPs expressed scepticism about the current delivery model of the CLI, pointing out that people often receive insufficient guidance throughout the programme. This concern led them to prefer offering personalised care or referring patients to a practice nurse or dietitian instead (*Q5, GP4*).

#### Insufficient physical activity support and programme limitations in the CLI for individuals with knee OA

Both individuals with knee OA and lifestyle coaches identified insufficient physical activity support as a major barrier to implementing the CLI for this group. Nearly all individuals with knee OA expressed disappointment, having expected group-based exercise sessions but instead receiving primarily theoretical information. They highlighted the importance of a structured exercise programme to strengthen knee muscles and alleviate OA-related pain (*Q6, CLI participant 6*).

Lifestyle coaches acknowledged the intervention’s knowledge-focused approach (*Q7, Lifestyle coach 4*) and emphasised the need for an integrated exercise component. However, they pointed to financial constraints under current reimbursement policies as a key obstacle (*outer setting, Q15, exercise professional 1*). Additionally, they noted that the programme’s name, *BeweegKuur* (‘*Movement Therapy’*), often misled participants about its scope (*Q8, lifestyle coach 6*).

#### Challenges in participation due to diverse knowledge levels and health literacy

Nine individuals with knee OA reported that the programme’s content was too superficial, leading to the drop-out of four out of these nine participants (*Q11, CLI participant 8*). Similarly, 10 individuals with knee OA felt that the group sessions did not resonate with them, citing differences in knowledge levels, age and comorbidities as barriers to participation (*Q12, CLI participant 5*). Lifestyle coaches echoed these concerns, noting the difficulty of tailoring group sessions to address participants’ varied backgrounds and health conditions (*Q13, lifestyle coach 1*).

Additionally, lifestyle coaches highlighted that the BeweegKuur programme struggles to meet the needs of individuals with inadequate health literacy, as it relies on self-management (*Q14, lifestyle coach 5*). While this issue was not reflected in our participant sample—none of whom had inadequate health literacy —three lifestyle coaches suggested that programmes like SSiB, with more hands-on support, might be better suited for disadvantaged neighbourhoods. However, GPs pointed out that referrals to such programmes depend on regional availability.

### Domain 2: outer setting

The second CFIR domain focuses on factors outside of the organisation affecting implementation. Key themes include financing (lack of reimbursement), local attitudes (sociocultural factors, social support), market pressure (scepticism among GPs), external policies (accessibility of unhealthy food, reimbursement for weight management medication) and partnerships (lack of care coordination).

#### Sociocultural factors and social support

One person with knee OA from a non-Western background reported high satisfaction and successful lifestyle changes (*Q16, CLI participant 3*). In contrast, two other individuals from non-Western backgrounds encountered difficulties in adjusting cultural eating habits. They mentioned difficulties in adapting traditional meals to healthier dietary choices while preserving cultural relevance (*Q19, CLI participant 18*). Lifestyle coaches recognised the importance of cultural norms and values in dietary and physical activity recommendations. They noted that people from non-Western backgrounds often face greater challenges in adapting their lifestyle, such as differences in food preferences, meal preparation and attitudes towards physical activity (*Q20, lifestyle coach 4*). A GP further highlighted that patients from non-Western backgrounds frequently experience barriers to participation in the CLI, particularly due to concerns that cultural eating habits are not adequately acknowledged or accommodated (*Q21, GP1*).

Social support also played a significant role in facilitating behavioural change. Individuals with knee OA and lifestyle coaches agreed on the importance of social support for success in the programme (*Q17, lifestyle coach 4*). 16 people with knee OA indicated that family and friends were essential in helping them adopt healthier habits, such as making healthier food choices or exercising together. Some individuals also found support in the group sessions, where they could learn from shared challenges and experiences (*Q18, CLI participant 19*). In contrast, seven people with knee OA reported being intrinsically motivated and did not require social support.

#### Scepticism among GPs regarding the implementation of the CLI

Three GPs expressed concern about the commercialisation of the CLI, criticising its reliance on a free-market model. They argued that this approach has led to uncertainty about the quality and consistency of care, which influenced their decision not to refer patients to the programme (*Q22, GP 5*).

### Domain 3: inner setting

The third CFIR domain addresses internal organisational factors and preparedness for implementation. Key themes include available resources (short lines with other HCPs, physiotherapist for physical symptoms; barriers: CLI offered outside the neighbourhood, limited consultation time, unsuitable locations, lifestyle coach inconsistency), compatibility (mismatched expectations) and relational connections (fit to coach).

#### Fit to coach

The majority of individuals with knee OA highlighted that a positive relationship with the lifestyle coach was a key facilitator for success in the CLI. Effective coaches were described as knowledgeable, passionate, accessible and non-judgmental (*Q38, CLI participant 15*). One person expressed regret after the programme ended, emphasising the value of the appointments with the lifestyle coach (*Q39, CLI participant 9*). Conversely, people reported barriers when the lifestyle coach was unresponsive to their individual needs (*Q40, CLI participant* 5) or communicated in a negative manner (*Q41, CLI participant 20*).

### Domain 4: individuals

The fourth CFIR domain examines how personal beliefs and factors influence the adoption and success of the innovation. Key themes include motivation (participant motivation and engagement in lifestyle change), need (importance of non-surgical options) and capability (lifestyle coaches’ expertise in OA support).

#### Participant motivation and engagement in lifestyle change

Most individuals with knee OA reported being motivated to improve their lifestyle and acknowledged understanding the steps required to improve their health (*Q42, CLI participant 5*). However, many mentioned struggling to translate this knowledge into action. Six individuals highlighted challenges with discipline and emphasised the need for accountability (*Q43, CLI participant 13*).

Lifestyle coaches underscored that motivation and self-management are critical for success in the programme, noting that the CLI primarily provides tools and is not suited for those with a passive attitude (*Q44, exercise professional 1*). Some GPs agreed with this view, while others argued that the CLI is better equipped to address motivational challenges compared with standard dietetics or physiotherapy. Two GPs stated that patients’ lack of motivation and subsequent dropouts negatively impacted their own motivation to refer patients to the CLI (*Q45, GP 6*).

#### Expertise among lifestyle coaches for OA support

The SSiB lifestyle coach emphasised the value of physiotherapy appointments in their SSiB programme, which allowed more tailored exercise plans based on specialised knowledge (*Q47, lifestyle coach 8*). On the other hand, the BeweegKuur exercise professional expressed confidence in designing exercise plans but noted that the single appointment limits opportunities for follow-up (*Q48, exercise professional 1*). Five BeweegKuur lifestyle coaches reported challenges in adapting these plans due to limited expertise, often requiring referrals to external physiotherapists (*Q49, lifestyle coach 5*). Additionally, one lifestyle coach pointed out that the BeweegKuur programme benefits from a multidisciplinary team, whereas lifestyle coaches in the CooL programme work independently as the sole providers (*Q50, lifestyle coach 7*).

## Discussion

This qualitative study identified determinants for implementing the CLI for individuals with knee OA and overweight, based on insights from both individuals with knee OA and HCPs. Key factors were explored across four CFIR domains: innovation, outer setting, inner setting and individuals. These findings highlight critical areas for improvement to better align the CLI with the needs of people with knee OA.

While the CLI is recognised as a promising treatment for people with knee OA and overweight, challenges in its design and adaptability significantly affected its implementation. Both HCPs and people with knee OA noted a disconnect between the programme’s performance and its potential, primarily due to the lack of an integrated exercise component aimed at improving knee function and overall health. Despite the programme’s name, *BeweegKuur* (‘Movement Therapy’), it does not include supervised exercise, a discrepancy noted in prior research, underscoring the need for greater transparency.^22^ Although the CLI addresses motivation through education, it falls short in providing the structure and guidance needed to translate intentions into action—revealing a classic intention-behaviour gap.^23^ Exercise is proven to alleviate pain, improve joint function and enhance quality of life for patients with OA. Integrating a tailored exercise component could better meet participants’ needs and improve long-term outcomes.^24 25^ However, lifestyle coaches reported that current reimbursement policies limit the inclusion of such components. Prior research indicates that the CLI reimbursement policies were implemented too quickly, leading to incomplete or poorly executed programme aspects,^24^ potentially fuelling GP scepticism about the quality of care. These challenges underscore the urgent need for policy reforms that ensure equitable reimbursement models focused on disease prevention and healthy lifestyle promotion.^26^,^28^

Lifestyle coaches emphasised that self-management and motivation are key to success in the CLI. While most participants felt motivated and understood the necessary steps to improve their health, many struggled to act on this knowledge, highlighting the intention-behaviour gap.^23^ Many HCPs may be unfamiliar with this concept, which can hinder effective weight management support. A lack of behaviour change is not necessarily a sign of low motivation but can stem from environmental barriers, fear of movement or insufficient guidance.^29^ Without a deeper understanding of these factors, HCPs may struggle to provide the support needed to help patients overcome these barriers.

A gap in lifestyle coaches’ expertise in adapting exercise plans for knee OA was identified. This aligns with earlier qualitative research in the Netherlands, which similarly highlighted a lack of HCP expertise and suboptimal organisation of care as barriers to non-pharmacological OA treatment.^27^ This issue extends beyond the Netherlands, as international studies also emphasise the need for HCP education on integrating physical activity into OA management and weight loss programmes.^30^,^34^ Developing tailored exercise protocols for OA could help HCPs provide more targeted treatment.^35^ Additionally, lifestyle coaches in this study highlighted the importance of physiotherapists in multidisciplinary teams, supporting evidence that such interventions improve functional outcomes and patient satisfaction.^27 36^ While patients with OA can be referred for exercise therapy during CLI participation, making physiotherapists a standard part of the programme could enhance efficacy by ensuring continuity of care and reducing treatment fragmentation.^37^

Many lifestyle coaches reported difficulties tailoring group sessions to participants’ varying knowledge levels, health literacy and health conditions and also highlighted a lack of motivation for lifestyle changes. A previous study similarly found that individuals with a deeper understanding of their health do not value generic weight management advice, particularly when it does not resonate with their specific circumstances.^38^ In our study, participants were more receptive to advice when they perceived their coach as knowledgeable, non-judgmental and empathetic, aligning with other studies emphasising the importance of strong participant-provider relationships in obesity treatment.^38^,^40^

Additionally, another study involving patients with chronic illnesses emphasised the importance of structural components within the CLI—such as the easy accessibility of the intervention site, the relevance of the programme content and the presence of HCPs during exercise sessions—as critical facilitators for programme adherence and outcomes.^28^ Similar factors emerged as key facilitators in our research. These insights suggest that effective implementation of lifestyle interventions in populations with chronic conditions requires both structural facilitators—such as accessible intervention sites and trained HCPs—and personalised support that addresses individual patient needs and literacy levels.

Cultural background and health literacy emerged as additional factors influencing the success of the CLI. Individuals from non-Western backgrounds faced barriers due to cultural eating habits that were not accommodated, despite lifestyle coaches’ efforts to tailor the programme. This highlights the need for culturally sensitive interventions.^41^,^43^ Additionally, the BeweegKuur programme’s reliance on self-management posed challenges for individuals with limited health literacy, as it requires health knowledge and autonomy.^44^ Nevertheless, two participants with marginal health literacy adapted their lifestyle, suggesting that the programme can be effective with adequate support. Prior research underscores the challenges faced by individuals with low socioeconomic status or non-Western backgrounds, emphasising the need for culturally relevant materials, literacy support and the involvement of community leaders to improve engagement and outcomes.^45^

### Implications for clinical practice and future research

The findings of this study highlight important implications for clinical practice and policy. To sustain the CLI in routine care for individuals with knee OA, programme adaptations—a process known as reintervention —are required.^23^ These include incorporating a tailored exercise component, improving the expertise of lifestyle coaches and establishing a fairer funding model. Building GPs’ trust through education and clear communication of programme outcomes could reduce their scepticism and facilitate better integration into primary care.^14 26^ Future research should focus on overcoming these barriers and using the facilitators identified in this study by developing and testing tailored implementation strategies through a pilot study. Additionally, evaluating the CLI’s reach, effectiveness, adoption, implementation and sustainability will be essential for its successful integration into routine care.

### Strengths and limitations

Earlier studies have explored barriers and enablers for lifestyle programmes, but to our knowledge, this is the first qualitative study examining the implementation of the CLI in Dutch primary care for patients with knee OA from both participant and HCP perspectives. Several methods were used to enhance the validity of the findings, such as using a solid research framework and independent coding of transcripts by two researchers. A key strength is that the findings emerged within the context of an RCT, allowing us to identify key insights prior to assessing (cost-)effectiveness and reducing interpretation bias. By including individuals with knee OA and HCPs from various CLI programmes outside the LITE trial and using purposive sampling, we ensured a diverse sample in terms of age, sex, level of education, health literacy, cultural background and session attendance.

However, several limitations should be considered. Since the study was conducted in the Netherlands, the findings may be less generalisable to other countries with different healthcare systems. Despite efforts to ensure diversity, the sample includes only one exercise professional and one lifestyle coach from other CLI programmes than BeweegKuur. Additionally, as most individuals with knee OA were recruited through the LITE trial, their motivation may differ from patients in standard care. In this study, we used educational level. This could be used as a proxy for socioeconomic position, acknowledging that this measure does not encompass all dimensions of socioeconomic position. Finally, interviewing participants in their final phase of, or after, the CLI may have introduced recall bias, though triangulating data from people with knee OA and different HCPs helped strengthen the validity of our results.

### Conclusion

The implementation of the CLI for individuals with knee OA is influenced by several key factors, including the intervention’s design, the organisational and policy context and individual characteristics. The identified factors underscore the necessity of refining the CLI to better align with participant needs, ultimately improving its effectiveness and long-term sustainability as a treatment strategy for individuals with knee OA and overweight.

## Supplementary material

10.1136/bmjopen-2025-108216 online supplemental file 1

## Data Availability

Data are available upon reasonable request.
