# Supplementary material for: Determinants for the implementation of a combined lifestyle intervention for patients with knee osteoarthritis and overweight: a qualitative study
Source: BMJ Open. 2026 Jan 28;16(1):e108216. doi: 10.1136/bmjopen-2025-108216 (PMC12853553; doi:10.1136/bmjopen-2025-108216)
Supplement: online supplemental file 1 [file bmjopen-16-1-s001.pdf]

Supplementary Files: Table S1 and interview guides

Supplemental file Table S1

Table S1: Overview of Recognized CLI Programs

CLI program	BeweegKuur	Samen Sportief in Beweging	SLIMMER	CooL	X-Fittt	Keer Diabetes2 Om	Keer Diabetes2 Om – GLI Online
**Overall aim**	The CLI is intended for people with overweight and obesity. It focuses on achieving and maintaining a healthy lifestyle.						
**Target group**	• People with obesity<br>• People with overweight who have a moderate to high health risk for chronic conditions						
**Specific Target Group**	No specific target group	Patients with limited health literacy and/or a low socioeconomic status	No specific target group	No specific target group	No specific target group	Patients with prediabetes or type 2 diabetes	Patients with prediabetes or type 2 diabetes
**Main Providers**	Lifestyle coach, dietitian, exercise professional	Lifestyle coach, dietitian, physiotherapist, local sports providers	Lifestyle coach, dietitian, exercise professional	Lifestyle coach	Lifestyle coach	Nurse, dietitian, coach	Nurse, dietitian, coach
**Core Components**	Nutrition, physical activity, sleep, relaxation, exertion	Nutrition, physical activity with local community	Nutrition, physical activity, sleep, stress, relaxation	Nutrition, physical activity, sleep, personal balance	Nutrition, physical activity, sleep, stress,	Nutrition, physical activity, sleep, relaxation	Nutrition, physical activity, sleep, relaxation

					alcohol, smoking		
**Physical Activity Sessions**	No	Yes	Yes	No	Yes	Yes	Yes
**Transition to Regular Exercise**	Yes, if collaborating with local sports providers	Yes, generally possible	Yes, generally possible	Yes, if collaborating with local sports providers	Yes, linked to an exercise center	No formal transition	No formal transition
**Delivery Format**	Physical	Physical	Physical + Digital version (SLIMMER by Ancora)	Physical + Digital version (MiGuide)	Physical	Physical	Digital

Supplemental file: Interview guides

Interview guide for individuals with knee OA

1. Osteoarthritis

- What was the main reason you decided to participate in the lifestyle program?
  - Can you tell me more about why [reason] played a role in your decision to participate?
- What did you hope to achieve with the program?
  - Can you describe whether your expectations aligned with your experiences in the program?
- Did you feel that you received support in managing your knee osteoarthritis during the lifestyle program?
  - How would you like to improve the support you received?

2. Relationship with Healthcare Provider

- How would you describe your relationship with your general practitioner (GP)?
- Have you ever talked to your GP about lifestyle or a lifestyle program?
- Do you feel comfortable communicating with your GP about the lifestyle program and your experiences with it?
  - What makes you feel comfortable or uncomfortable? Are there other healthcare providers or people with whom you share your experiences?
  - How would things have been different if your GP had been a man or a woman? In what way?
- How would you describe your relationship with your lifestyle coach (dietitian/physiotherapist) during the program? [If you had multiple lifestyle coaches, please specify which one you are referring to]
- Did your relationship with your lifestyle coach influence your participation and success in the program?

    - Was there something specific in the communication or behavior of your lifestyle coach that helped or hindered you?
    - How would things have been different if your lifestyle coach had been a man or a woman? In what way?
    - How would things have been different if your lifestyle coach shared the same cultural background as you?

3. Social Support

- Who provided you with support in your decision to participate in the lifestyle program and during your participation in the program?
  [Only if the participant asks for clarification: "Think of family members, friends, colleagues, or others in your social circle."]
    - How did this person support you?
    - Was this support helpful or not? And why or why not?
    - In what ways do these people help you reach your goals or make lifestyle changes?

4. Program Process and Challenges

Barriers

- Did you experience any barriers or challenges during the program? If so, what were they?
  [If the question is unclear, provide clarification: “Was there anything specific that was hard to stick to, or that prevented you from achieving your goals?”]
    - Can you explain the reasons why these aspects were experienced as obstacles or challenges?
    - How did you deal with these obstacles?

Specific Factors:

- How are roles divided at home? Did your gender (male/female) influence the division of roles at home?
- How did caregiving responsibilities impact your participation in the program? [Think about tasks such as caring for children or elderly family members]

- Do you think gender plays a role in these caregiving responsibilities?

- How did your work situation impact your ability to follow the program? Was the program manageable alongside work?
  - Do you think gender plays a role in this?
- Could you describe the primary caregiving tasks? [Think about caregiving for children, work-life balance, social network, stigmatization, or other gender-related roles].

Facilitating Factors:

- What helped you stay motivated and engaged during the program?
  - Can you explain why these factors helped encourage your participation?
- Did you create a plan together with your lifestyle coach? [If no, do not ask further].
  - What did the plan look like? How well did you stick to the plan?
  - Was the program carried out according to the plan? [If yes] Can you describe how? [If no] Why not?
- How did the group sessions go? [If no, why not?] [If yes, could you tell me more about the group (e.g., composition of the group)?]
- Are there aspects that you think were unnecessary in the program or that should be added? If yes, which ones?
- Can you describe whether you feel confident in your ability to maintain a healthy lifestyle after completing the program?
  - What gives you that confidence (or lack thereof)?
  - How do you plan to continue the changes to your healthy lifestyle after the program ends?
- Did you receive information about the lifestyle program?
  - Was the information clear?
  - Did you review the information with others?
  - What information did you miss? What additional information would you need?

5. Culture/Socioeconomic Status/Health Literacy

- We know that cultural background can influence choices regarding lifestyle and nutrition.
  I will give a few examples. In some cultures, eating is a social activity, while in others it is seen as a necessity. Another example is that in some cultures, it is important to join sports clubs. How does this apply to you?
  - How has your cultural background (or that of your lifestyle coach) possibly influenced your decision to participate in the program [and your experiences during the program]?
- Can you describe to what extent financial factors played a role in achieving your goals and maintaining lifestyle changes?

Interview guide for General Practitioner (GP)

- What is your role within your organization and what role do you play in lifestyle interventions?
- Are you satisfied with your role in offering lifestyle interventions in the care of people with knee osteoarthritis? If not, what role would you like to have?

1. **Knowledge and Beliefs**
   - To what extent is the CLI embraced within your practice?
     - Are regular meetings held in your practice? With whom?
     - Is the CLI discussed during these meetings?
     - What is discussed?
   - What information about the CLI has been made available to you?
   - Who do you ask if you have a question about the intervention or its implementation?
     - How accessible are these individuals?
2. **Osteoarthritis**

- What is your view on the implementation of combined lifestyle interventions for people with knee osteoarthritis?
- Are you convinced that combined lifestyle interventions are effective for patients with knee osteoarthritis?
  - Why or why not? / Why do you think the CLI is an effective intervention for patients with knee osteoarthritis and overweight?
- How does the CLI compare to other treatment methods (e.g., painkillers, knee injections) in terms of effectiveness for people with knee osteoarthritis and overweight?

3. **Patient Needs**
   [Check with the questionnaire whether the GP has referred patients to the CLI.] If not, why? If yes, proceed with the following questions:
   - How do patients with knee osteoarthritis respond when you refer them to the CLI?
   - Is there another treatment method that patients with knee osteoarthritis and overweight would prefer?
     - [If yes] Could you describe this treatment method? Why would patients prefer this alternative?
   - Have you encountered any specific challenges when offering the CLI to your patients?
     - [If yes] Could you name some of these challenges?
     - How does the socioeconomic status of a patient affect your decision to refer them to the CLI?
     - Have you faced challenges in referring patients from different cultural backgrounds?
     - Do different levels of health literacy affect your decision to refer a patient to the CLI? If yes, how?
   - On the other hand, have there been any factors that have promoted the offering of the CLI?
4. **Gender and Sex**

- Are you aware of any gender or sex-related factors that could affect the lifestyle intervention and your relationship with your patient? Have you ever noticed challenges when the participant is of a different gender than you?
- Do you consider any challenges when the participant is of a different gender than yourself?

5. **Collaboration with Healthcare Providers**
   - How have you experienced collaboration with other healthcare providers, such as the lifestyle coach?
   - What is your opinion on the attitude of the involved healthcare providers towards the CLI?
6. **Referral/Implementation**
   - What barriers do you see for the further implementation of the CLI for people with knee osteoarthritis?
   - What conditions do you think are necessary for the successful implementation of the combined lifestyle intervention for people with knee osteoarthritis?
   - What actions should be taken to refer more patients with knee osteoarthritis to the CLI?

Are there any other important aspects you would like to emphasize regarding your role as a GP in lifestyle interventions?

Interview guide for Lifestyle Coach

- What is your role within your organization?
- Could you tell me more about your role in lifestyle interventions?

1. **Knowledge and Beliefs**
   - What do you think are the most important aspects to consider when providing the CLI?

- Are you convinced that the CLI is effective for participants with knee osteoarthritis and overweight?
  - Why or why not? / Why do you think the CLI is an effective intervention for patients with knee osteoarthritis and overweight?

2. **Osteoarthritis**

- How do you adapt the CLI for people with knee osteoarthritis? And for people with other conditions?
- Has the fact that the participant has knee osteoarthritis made providing the lifestyle intervention more difficult or presented challenges? If yes, in what way(s) have you experienced this? (Possibly compared to other conditions within the CLI)
- Did you feel sufficiently competent to account for participants who have knee osteoarthritis when offering the lifestyle intervention?
  - Did you receive any information/training to offer the lifestyle intervention to participants with knee osteoarthritis?
- What barriers do you see for the implementation of the CLI for people with knee osteoarthritis?
- What factors can promote the implementation of the CLI for people with knee osteoarthritis?

3. **Participants**

- How would you describe the relationship with the participants during the program?
- In what ways do you ensure this relationship is established?
- To what extent do you consider it important that the participant has a strong social network for the success of the intervention?
- How do you ensure that this is established?
- Are there participants who are financially struggling, making lifestyle changes difficult? Could you provide an example?

    - How do you support participants in finding affordable alternatives for healthy lifestyle choices?
  - Can you describe the cultural diversity among the participants of the lifestyle program?
    - Do you think cultural backgrounds influence participants' eating habits and food preferences? If yes, how? And their physical activity?
  - How do the educational level and health literacy of participants vary?
    - Do you adjust how you convey information to this? Could you give an example of how you adjust communication?
    - Do you think a low education level and/or low income affects participants' eating habits and food preferences? If yes, how? And their physical activity?
  - Are you aware of any gender or sex-related factors that could influence the lifestyle intervention and your relationship with your patient?
  - How do you handle any challenges when the participant is of a different gender than yourself?

4. **Collaboration with Healthcare Providers**
  - How do you experience the collaboration with other healthcare providers involved in the care of the participant? For example, the GP.
  - What is your opinion on the attitude of the involved healthcare providers towards the CLI?

5. **Implementation Process**
  - Was a plan developed within the organization to implement the CLI for people with knee osteoarthritis and overweight? If no, do not follow up. If yes, proceed with the following questions:
    - Can you describe the plan?
    - What was your role?
    - Who was involved? What were their roles?
  - When do you consider the implementation to be successful?

- Do you receive feedback reports/evaluations about delivering the CLI?
    - [If no] Would you like to receive them and what would you want to see?
    - [If yes] What do they look like? How useful are these reports? How could they be improved? How often do you receive them? Where do they come from?
- What is the communication strategy of the organization to promote the CLI? And specifically for people with knee osteoarthritis?
- What would you advise other healthcare professionals who want to implement the CLI for people with knee osteoarthritis?

Are there any other important aspects you would like to emphasize regarding your role as a lifestyle coach (dietitian/exercise professional) in lifestyle interventions?

Supplemental Table S2: Detailed characteristics participants

Supplemental Table S2: Detailed characteristics participants

ID CLI Participant	gender	Age category	Cultural background	Level of education	CLI status	Health literacy status
1	Woman	65-69	Dutch	Low-Middle	Stopped	Adequate
2	Man	45-49	Dutch	High	Stopped	Adequate
3	Woman	55-59	Surinam	Low	Completed	Marginal
4	Man	55-59	Indo-Dutch	Low	Stopped	Adequate
5	Woman	45-49	Dutch	Low-Middle	Completed	Adequate
6	Woman	60-64	Dutch	Low	Completed	Adequate
7	Man	45-49	Dutch	Low-Middle	Completed	Marginal
8	Woman	45-49	Dutch	High	Completed	Adequate
9	Man	65-69	Dutch	Low	Completed	Adequate
10	Woman	50-54	Surinam	Low-Middle	Completed	Adequate
11	Woman	60-64	Dutch	High	Completed	Adequate
12	Woman	50-54	Dutch	Low	Ongoing	Adequate
13	Woman	50-54	Surinam	Low-Middle	Ongoing	Adequate
14	Man	60-64	Unknown*	High	Completed	Adequate
15	Woman	55-59	Unknown*	High	Completed	Adequate
16	Woman	45-49	Dutch	Low-Middle	Completed	Adequate
17	Woman	60-64	Ducth	Low	Stopped	Adequate
18	Woman	60-64	Surinam	High	Ongoing	**
19	Man	55-59	Dutch	Low-Middle	Stopped	Adequate
20	Woman	55-59	Dutch	High	Completed	Adequate
21	Man	70-74	Ducth	Low	Ongoing	**

22	Woman	50-54	Dutch	Low-Middle	Ongoing	**
23	Woman	55-59	Dutch	Low-Middle	Ongoing	**

**Health care professional**	**gender**	**age**	**Background**	**SEP[#] of practice**
GP^ 1	Woman	Unknown	Dutch	Unknown
GP 2	Man	55-59	Dutch	High
GP 3	Woman	35-39	Dutch	High
GP 4	Man	Unknown	Dutch	Unknown
GP 5	Woman	35-39	Dutch	Low-Mixed
GP 6	Man	50-54	Dutch	High-mixed
GP 7	Woman	40-44	Dutch	Mixed
LSC^^ 1	Woman	Unknown	Dutch	NA
LSC 2	Woman	Unknwon	Dutch	NA
LSC 3	Woman	45-49	Dutch	NA
LSC 4	Woman	55-59	Dutch	NA
LSC 5	Woman	30-34	Western (one parent from Turkeye)	NA
LSC 6	Man	35-39	Non-western (not specified)	NA
LSC 7	Woman	Unknown	Dutch	NA
LSC 8	Woman	35-39	Dutch	NA
Exercise professional	Woman	30-34	Dutch	NA

* not born in the Netherlands but unknown how they would indicate their cultural background; ** missing data; ^GP = general practitioner; ^^ = Lifestyle coach; NA = not applicable; [#] SEP = socioeconomic position of patient population of practice

Supplemental Table S3: Summary of findings by the Consolidated Framework for Implementation Research domains, constructs and corresponding themes

Table S3: Summary of findings by the Consolidated Framework for Implementation Research domains, constructs and corresponding themes

Innovation domain	Outer Setting Domain	Inner Setting Domain	Individuals Domain
**1. Relative Advantage / theme: Perceived effectiveness of the CLI for OA management**<br><br>Facilitator:<br><br>*"I hardly have any complaints anymore. I've lost some weight—quite a few kilos, actually. And what I experience now when I walk is nothing like what I used to feel before. I no longer feel as if my knee is locking up."* ***(Q1, CLI participant 9)***<br><br>*"But I have to say, the people with knee complaints that I speak to, they do have less pain. So I think that's really good. And whether that's because of the weight loss or the exercises they get, I can't really give an answer to that."* ***(Q2, Exercise professional 1)***<br><br>*"When you look at complaints with OA, whether it's the knee or any other type of OA, the CLI can certainly help. For example, it can prevent someone from ultimately needing a prosthesis. And that's a very positive thing in my view, if you don't have to operate. So, there's definitely potential there,*	**4. Financing**<br><br>Barrier:<br><br>*"Look, these people should actually be exercising, preferably with some guidance. But well, you can't expect that from a program that's reimbursed by health insurance. That's not possible. I understand that people really want that, but that's just not feasible."* ***(Q15, Exercise professional 1)***<br><br>**5. Local attitudes / theme: Sociocultural factors and social support**<br><br>Facilitator:<br><br>*"My parents had a small grocery store in Surinam. We would wake up, eat, I sometimes had bread with a bottle of coke. We'd get some money, go to school, eat bread, drink coke, or have noodles with coke —just coke, coke, coke. That's how we grew up, and here we are, turning 60. And now, here in the Netherlands, they say you have to do*	**9. Available Resources**<br><br>Facilitator:<br><br>*"We had multiple external physiotherapists, and no one really knew exactly which physiotherapist was assigned to which patient. It was a complete mess, so now that we have this managed internally, it just brings a lot more peace."* ***(Q29, Exercise professional 1)***<br><br>*"In the beginning, it was a bit confusing. How does the referral process work? And once we referred them, where did they go? We'd lose track of them—Now we have a more short lines for follow-up, and it's organized much better."* ***(Q30, GP 7)***<br><br>*"I think it mainly comes down to that part of movement, that they really need to be properly guided in that. So, I believe that what we already have with the*	**12. Motivation / theme: Participant motivation and engagement in lifestyle change**<br><br>Facilitator:<br><br>*"And then you hear it's early-stage OA, and I thought, well, I'd really like to do whatever I can to adjust my lifestyle so that, if possible, it doesn't get worse any faster."* ***(Q42, CLI participant 5)***<br><br>Barrier:<br><br>*''If I just get sent back home with exercises. Yeah then you know. You don't have the motivation to do those exercises every day again.''* ***(Q43, CLI participant 13)***<br><br>*"You can really tell that, in this program, the ball is in their court. They really have to take action. And if people have a passive attitude, which a lot of people do, then the program doesn't fully*

*but not with the current approach.'' **(Q3, GP4)***<br><br>Barrier:<br><br>*"She asked, 'So, what are you doing?' And, well, I said, 'I'm trying to live as healthily as possible. I have one vice, which is that I drink wine. But other than that, we live as healthily as we can and stay as active as possible.' After talking like this for a while, she said, 'I don't think there's anything I can add.'" **(Q4, CLI participant 1)***<br><br>*"I'm quite negative about it. I have much more confidence when I see people myself, or through the nurse practitioner, who can provide certain services, like dietitian advice, and if necessary, a referral to dietetics. But this approach creates a certain expectation for people, which ultimately only leads to demotivation. It doesn't reduce knee OA at all. People won't change their habits—they won't start exercising, at least not in my experience—because they're not being motivated to do so." **(Q5, GP4)***<br><br>**2. Design / theme: Insufficient physical activity support and program limitations in the CLI for individuals with knee OA**<br><br>Barrier:<br><br>*"I expected that we would be doing exercises under the supervision of a physiotherapist—*	*this and that to live healthily. If I have to be honest, my whole life has improved a lot. I learned a lot of things from the lifestyle coach." **(Q16, CLI participant 3)***<br><br>*"My experience is that when people are supported by their family at home, it goes much more easily. Those who also have to go against the flow at home, of course, find it much more difficult." **(Q17, Lifestyle coach 4)***<br><br>*"The group discussions with the people are definitely pleasant. You're talking to others who are in the same situation, so you know you're not alone—that's one important aspect." **(Q18, CLI participant 19)***<br><br>Barrier:<br><br>*''As a Hindu, I also told my lifestyle coach that I love eating rice. If you think I am going to leave my rice, no way! I will never do that.'' **(Q19, CLI participant 18)***<br><br>*"You often notice that people find it very difficult to break away from a certain culture or habits." **(Q20, Lifestyle coach 4)***<br><br>*"For people with a non-Western background, the threshold is higher to even begin. There's also some fear that their own eating habits, shaped by their*	*physiotherapist is great, but you shouldn't add a fitness instructor to the CLI program, you should really have a physiotherapist. Because the physiotherapist can take the physical complaints into account." **(Q31, Lifestyle coach 8)***<br><br>Barrier:<br><br>*"It definitely worked better when it was still offered here in the neighborhood, but unfortunately, that stopped a few years ago. Now, we notice that if we want to offer it to people and they realize they'll have to travel outside the neighborhood, there's already resistance" **(Q32, GP 1)***<br><br>*"When someone comes in, often, it's not just with one complaint and overweight or obesity. You then have to bring up the idea of joining the CLI. And that takes time too, right? Because they'll ask, 'Oh, what's that?' Then you have to explain, which can easily take another 10-15 minutes. Meanwhile, you only have 10-15 minutes in total*	*align." **(Q44, Exercise professional 1)***<br><br>*"I think from my own practice, we've had a handful of people start, but all of them dropped out after a year or half a year. They find it difficult to stick with it, so because of that, I'm not very motivated to keep referring people in that direction, because I really question whether it helps." **(Q45, GP 6)***<br><br>**13. Need**<br><br>Facilitator:<br><br>*"I've started another CLI program [Xfittt] because I genuinely believe in it. I really don't want a gastric bypass or anything like that. This is really what I was looking for and what I had hoped to find in the BeweegKuur program." **(Q46, CLI participant 11)***<br><br>**14. Capability / theme: Expertise among lifestyle coaches for OA support**<br><br>Facilitator:<br><br>*''I believe that instead of involving a fitness instructor in*

exercises that would strengthen and improve my knee, and overall reduce the discomfort in my knee. And that, of course, was the only thing that was really important to me, but it wasn't addressed." ***(Q6, CLI participant 6)***<br><br>*"It is a very verbal and knowledge-oriented intervention. With the implementation of CooL, I quickly realized that there is a big gap between knowing what to do and being able to do it. So, it's a gap between knowledge and skills, and I also noticed that many people, at least the ones in my group, do possess that knowledge."* ***(Q7, Lifestyle coach 4)***<br><br>*"I think that the people from the LITE study don't feel completely heard. At least, that's been the case with this CLI, because this CLI doesn't necessarily include movement, it is only called BeweegKuur (Movement Cure)."* ***(Q8, Lifestyle coach 6)***<br><br>*"Well, the duration of the CLI, the fact that it lasts so long, is something I always get feedback about from my participants, but I personally also think it's much, much too long. Two years, that means the sessions are too far apart, and people lose touch with the program."* ***(Q9, Lifestyle coach 4)***<br><br>*"I do think the CLI is too short. OA often occurs later in life, so people have lived a certain way for 40, 50, or even 60 years. You*	*cultural background, won't be taken into account. However, this doesn't have to be the case at all. I know that there are many dietitians who specialize in different cultural dietary habits. So, it often requires more effort to make this accessible to them."* ***(Q21, GP 1)***<br><br>**6. Market Pressure / theme: Skepticism among GPs regarding the implementation of the CLI**<br><br><u>Barrier:</u><br><br>*"I have the feeling that it has become a kind of cash cow for companies that are capitalizing on a gap in the market, making a lot of money from it, and then not delivering the product they promise. So far, there is not a single provider that we trust to do their job well and offer the CLI."* ***Q22, GP 5)***<br><br>**7. External policies & Incentives**<br><br><u>Barrier:</u><br><br>*"Well, actually, I'm in favor of them giving away vegetables and fruit for free, and multiplying the price of chips and cookies tenfold, so to speak. Whereas now, they're often on sale, like 'buy 6, pay for 5,' or 'buy 4, pay for 3.' So, you're more likely to grab that unhealthy junk."* ***(Q23, CLI participant 23)***	*to address their primary complaint in the first place."* ***(Q33, GP 4)***<br><br>*"Yeah, and the funny thing was, all the people participating obviously had knee problems, and then it was on the third floor. Some participants really couldn't even make it up the stairs."* ***(Q34, CLI participant 6)***<br><br>*"What was frustrating during the entire process was that I saw three or four different lifestyle coaches because someone left, got sick, or resigned. And every time, I had to tell my story again. It felt like starting over each time.''* ***(Q35, CLI participant 8)***<br><br>**10. Compatibility**<br><br><u>Barrier:</u><br><br>*"Many patients think the program is focused on the knee, but the connection with weight loss is often overlooked. They assume the focus will be on the knee itself or that the knee will be 'fixed.'"* ***(Q36, Lifestyle coach 6)***	*the CLI, you really need a physiotherapist. We already have that. A physiotherapist can take the physical complaints into account. We provide them with a list: 'These are the people you'll be working with, and these are the complaints they have.' Then they can suggest alternative exercises tailored to those specific complaints.''* ***(Q47, Lifestyle coach 8)***<br><br><u>Barrier:</u><br><br>*"When they come to me for exercise advice, we first talk about how to strengthen the muscles around the knee joint. The most important thing is that the movement is gradually increased, and they need to start at a level that's achievable for them. Having an evaluation moment, maybe in the second year, specifically for physical activity, would be really valuable. Because I often hear people say, 'Oh yes, this has really helped me a lot,' and then they're ready to progress further. But that's not always possible.''* ***(Q48, Exercise professional 1)***

can't completely change that in one or two years. It really needs more time to have a lasting effect." ***(Q10, GP 1)***<br><br>**3. Adaptability / theme: Challenges in participation due to diverse knowledge levels and health literacy**<br><br><u>Barrier:</u><br><br>*"It all stays on a very superficial level. They tell you things about nutrition, but I feel like, yeah, I already know all that. And a lot of it I'm already doing."* ***(Q11, CLI participant 8)***<br><br>*''In that group, there were people from a variety of backgrounds. I don't like using that word, but unfortunately, it exists. People came from different backgrounds, had different jobs, and varied intellectual levels. That's why I believe it's really important to focus on the individual rather than the group. For instance, there were people whose income was also a significant factor."* ***(Q12, CLI participant 5)***<br><br>*''I think if you were to put people with knee OA in the same group, it would improve, but then you would also like to have people with the same level of intelligence and with the same age together, so that is very difficult. It is something we struggle with in the CLI.''* ***(Q13, Lifestyle coach 1)***	*"Two other people were there because they were prescribed medication, Saxenda or something, to help them lose weight. They had to follow the program, otherwise, they wouldn't get the medication. But they were only there for that reason, not because they thought the CLI would benefit them.''* ***(Q24, CLI participant 12)***<br><br>*"Since the introduction of Saxenda, many people are only participating in the CLI to eventually get it. So, you have a group of people who are actually just waiting to participate for a year and then expect a quick fix with Saxenda, which is really quite a shame."* ***(Q25, Lifestyle coach 3)***<br><br>*"In a broad sense, I think we eat unhealthily in the Netherlands. There are too many unhealthy products in stores that are quite cheap. So, if we really want to make a change, it needs to come through politics and the food industry. We also need to become a bit functionally angry about it. A lot of the obesity problem stems from this, of course."* ***(Q26, GP 2)***<br><br>*"A portion of the patients participate mainly because they hope to eventually receive certain medication for weight loss. And then I think, we're all putting in*	*"I think the current way of offering it in the form of the CLI absolutely does not align with the, let's say, objectives that were set beforehand, and you can see that reflected in the results, or actually, in the lack of them."* ***(Q37, GP 4)***<br><br>**11. Relational Connections / theme: Fit to coach**<br><br><u>Facilitator:</u><br><br>*''A good lifestyle coach is very important. That it is someone who does their job with passion. That someone listens to you and doesn't judge. Maybe that's a nice one too, not judging. I think that's also very important.''* ***(Q38, CLI participant 15)***<br><br>*''It's actually a pity it's over. They were always more or less fun outings. Conversations about what could be done better and how you could do things differently. That was always a lot of fun and we laughed a lot together. And every weight reduction goal that I set with him on every visit was successfully met.''* ***(Q39, CLI participant 9)***	*"Look, I've had situations where people said to me, 'I would really like to get specific exercises from you.' But I'm not an expert in nutrition or exercise, I am more of guiding and coaching people through the process. So at that moment, I had to explain that I didn't know. So with a training on OA, you might feel more confident during conversations."* ***(Q49, Lifestyle coach 5)***<br><br>*''A lifestyle coach doesn't necessarily need to be medically trained, whereas the BeweegKuur program includes a lifestyle coach and both a dietitian and a physiotherapist or exercise coach, which, to me, makes it stronger by being more multidisciplinary. The CooL program lacks this feature, meaning you are with a lifestyle coach for the entire time."* ***(Q50, Lifestyle coach 7)***

‘’I don't think BeweegKuur fits well in disadvantaged neighborhoods. You very much want someone to make a change on their own initiative, so that would always be the approach, but in practice you just see that it doesn't always work out for them.’’ ***(Q14, Lifestyle coach 5)***	*effort, thinking about it, and investing money, but what are we really doing this for in the end?"* ***(Q27, GP 4)***<br><br>**8. Partnerships & Connections**<br><br>Barrier:<br><br>*"Orthopedic surgeons will definitely mention that you need to lose weight, but if you just say it in one sentence, 'You need to lose weight, and that's it, good luck,' versus actually saying, 'Well, there’s a program, here’s a brochure, take a look and see what’s available near you,' I think that makes a huge difference. So, I think when it comes to knee OA, where you want to raise awareness the most, I think you're looking at orthopedic surgeons."* ***(Q28, GP 3)***	Barrier:<br><br>*"The dietitian I was assigned to just wasn’t a good fit, there was no real connection. She didn’t seem open to the person sitting in front of her—it was very much, ‘I’m the dietitian, and this is how it has to be, end of discussion."* ***(Q40, CLI participant 5)***<br><br>*"I never attended the physio sessions because I simply couldn’t make it. I was informed on such short notice, and it couldn’t be rescheduled. Then I got an email ‘If you’re not motivated, blah blah,’ and I just thought, what’s this about? The tone felt so aggressive that I thought, I’m not going there at all."* ***(Q41, CLI participant 20)***	
