# Supplementary material for: Determinants for the implementation of a combined lifestyle intervention for patients with knee osteoarthritis and overweight: a qualitative study
Source: BMJ Open. 2026 Jan 28;16(1):e108216. doi: 10.1136/bmjopen-2025-108216 (PMC12853553; doi:10.1136/bmjopen-2025-108216)
Supplement: Supplementary file 2 [file bmjopen-2025-108216.reviewer_comments.pdf]

PEER REVIEW HISTORY

BMJ Open publishes all reviews undertaken for accepted manuscripts. Reviewers are asked to complete a checklist review form and are provided with free text boxes to elaborate on their assessment. These free text comments are reproduced below.

ARTICLE DETAILS

Title (Provisional)

Determinants for the implementation of a combined lifestyle intervention for patients with knee osteoarthritis and overweight: a qualitative study

Authors

Gharbaran, Priya; Jansen, Nuria EJ; Merkelbach, Inge; van Middelkoop, Marienke; Schiphof, Dieuwke

---

VERSION 1 - REVIEW

Reviewer 1

Name Ji, Hong

Affiliation Nursing department, The First Affiliated Hospital, Shandong First Medical University

Date 19-Sep-2025

COI None

Overall, this qualitative study is well-structured and demonstrates clinical relevance.

However, I recommend the following revisions:

1. Study Sample: Please explain the rationale for achieving data saturation or sample size saturation in the study.

2. Data collection: Please supplement the interview framework for the semi-structured interviews.

3. The research themes and subthemes would be more clearly presented if illustrated with logical diagrams.

4. Please provide details on whether ethical approval was obtained prior to the study.

Reviewer 2

Name Pavlovic, Natalie

Affiliation South Western Sydney Local Health District Fairfield Hospital Library, Physiotherapy

Date **01-Nov-2025**

COI **None**

Reviewer comments

Thank you for an interesting qualitative study exploring the factors influencing the implementation of a combined lifestyle intervention for patients with knee osteoarthritis and overweight. I have some comments for consideration.

ABSTRACT

1. Under the 'design' subheading please state that this is a qualitative study.

2. Under the 'strengths and limitations' section, the abbreviation LITE is used for the first time (page 3 of the manuscript). Please type out the abbreviation in this section.

3. Under the 'what is already known on this topic' section it is stated that "the implementation of a Combined Lifestyle Intervention for this specific patient group in primary care remains poorly understood". However, there are existing qualitative studies exploring the barriers and enables to such programs for this patient group. For example:

• Lawford BJ, Bennell KL, Jones SE, Keating C, Brown C, Hinman RS. "It's the single best thing I've done in the last 10 years": a qualitative study exploring patient and dietitian experiences with, and perceptions of, a multi-component dietary weight loss program for knee osteoarthritis. Osteoarthritis Cartilage. 2021 Apr;29(4):507-517. doi: 10.1016/j.joca.2021.01.001. Epub 2021 Jan 9. PMID: 33434629.

• Barriers and enablers to the implementation of the Australian Osteoarthritis Chronic Care Program (OACCP). Eyles, J.P. et al. Osteoarthritis and Cartilage, Volume 28, S446.

• Wallis JA, Ackerman IN, Brusco NK, et al. Barriers and enablers to uptake of a contemporary guideline-based management program for hip and knee osteoarthritis: A qualitative study. Osteoarthr Cartil Open. 2020;2(4):100095. Published 2020 Aug 26. doi:10.1016/j.ocarto.2020.100095

METHODS

1. Can a brief description of the combined lifestyle intervention be provided as part of an appendix / supplementary file? The appendix could also acknowledge all of the combined lifestyle interventions that participants were recruited from (if that information is available).

2. Under the 'study design and conceptual framework' section please add a sentence stating that the data are reported using the Standards for Reporting Qualitative Research (SRQR) criteria and include a reference for this criteria.

3. Please add a sentence in the manuscript on whether there were any exclusion criteria when recruiting participants for this study.

4. Please comment in the manuscript on the background of the interviewers to ensure transparency. For example, are they experienced qualitative researchers? Do they have a specific clinical background?

5. Please include your interview guide either as part of your manuscript (e.g. as a table or figure) or the appendix for transparency.

6. Please add a sentence in the manuscript on whether pilot interviews were conducted to test the interview guide.

7. Please add a sentence in the manuscript on whether any repeat interviews were undertaken.

8. Please add a sentence in the manuscript on whether transcripts were returned to participants for comment and/or correction (i.e. member checking).

9. Under the 'data analysis' section, the author describes the coding process then states, "descriptive summaries were created for each theme to draw conclusions" (page 9). Please elaborate how the research team developed themes from the codes. For example, were themes formed by linking related codes then mapped onto components/constructs of the CIFR domains?

10. The methods sections should include a brief paragraph explaining how the researchers ensured the rigour of the findings (credibility, transferability, dependability and confirmability).

RESULTS

1. Please comment in your manuscript whether anyone approached for interview declined to participate.

2. In table 1, the socioeconomic status is determined by the participants' highest level of education obtained. However, there may be participants with lower levels of education who have higher socioeconomic status. It may be more appropriate to list the highest level of education attained by your participants in the table instead.

3. Adding to the above point, in the methods, it is stated that employment status was collected. Can this information be included in table 1 as well? For example, employed, unemployed, retired etc.

4. In table 1, 'Caucasian', 'Asian' and 'Mixed/multiple' are listed as ethnicities. However, these are not ethnicities. Examples of ethnic groups include Dutch, English, French, Chinese, Vietnamese, Assyrian etc. Please include the participants' ethnicities in the table.

5. In table 1, the healthcare professionals' occupations are listed as 'Lifestyle Coach/Dietitian' (n=8) and 'Exercise professional' (n=1). Later in the text it is stated that nine lifestyle coaches were interviewed. Can you please update your table to accurately reflect this? For example, it can be recorded as lifestyle coach (dietitian) and lifestyle coach (exercise professional).

6. In table 1 can you also clarify what is meant by ‘exercise professional’? For example, is it a physiotherapist, exercise physiologist or personal trainer?

7. Do you have data on the mean BMI and range in the patient group? If so, can it be included in Table 1?

8. Can an additional table be included outlining the characteristics for each individual patient participant? It can include their sex, age, BMI, education level, employment status, ethnicity/cultural background, attendance rate. This will provided context to the quotes provided by participants in your results section.

9. Please comment on whether the participants provided feedback on the findings (i.e. member checking).

10. Complexity is listed as one of the themes in your study. However, the quotes provided by the participants in Table 3 for ‘complexity’ reference the duration of the program which is more related to the ‘design’ theme. Can you please provide quotes that are more relevant to the ‘complexity’ theme?

11. I notice there are similarities between the themes listed. For example, under the innovation domain, the ‘design’, ‘adaptability’ and ‘complexity’ themes are all related to the design of the CLI program. Can these be grouped as one theme?

If the themes must remain separate and named according to the constructs in the CIFR, the data analysis section in the methods needs to describe this approach (see my comment under the methods section).

12. As I continue to read the manuscript, I can see themes in the text are named differently to the CFIR themes listed in Table 2.

a) Based on what I have read your themes are as follows:

Innovation domain

Theme:

• Perceived effectiveness of the CLI for OA Management

• Insufficient physical activity support and program limitations in the CLI for individuals with knee OA

• Challenges in participation due to diverse knowledge levels and health literacy

Outer setting domain

Theme:

• Sociocultural factors and social support

• Scepticism among GPs regarding the implementation of the CLI

Inner setting domain

Theme:

• Fit to coach

Individuals domain

Theme:

• Participant motivation and engagement in lifestyle change

• Expertise among lifestyle coaches for OA support

b) Table 3 should be amended so that the themes in the manuscript are listed under the CFIR domains. The participant quotes should correspond to your themes in the table.

c) Table 2 should then be amended. Instead of ‘CFIR theme’ it should read ‘CFIR construct’. Instead of ‘definition of theme’ it should be ‘definition of construct’. If appropriate, you may also add an additional column (e.g. named ‘corresponding theme’) to list the themes that are mapped to that domain / construct.

DISCUSSION

1. Under the ‘strengths and limitations’ section it is stated that this is the first qualitative study examining the implementation of the CLI for patients with knee OA from both participant and HCP perspectives. However, there are existing qualitative studies exploring the barriers and enables to such programs from patients and HCPs. For example:

• Lawford BJ, Bennell KL, Jones SE, Keating C, Brown C, Hinman RS. "It's the single best thing I've done in the last 10 years": a qualitative study exploring patient and dietitian experiences with, and perceptions of, a multi-component dietary weight loss program for knee osteoarthritis. Osteoarthritis Cartilage. 2021 Apr;29(4):507-517. doi: 10.1016/j.joca.2021.01.001. Epub 2021 Jan 9. PMID: 33434629.

• Barriers and enablers to the implementation of the Australian Osteoarthritis Chronic Care Program (OACCP). Eyles, J.P. et al. Osteoarthritis and Cartilage, Volume 28, S446.

• Wallis JA, Ackerman IN, Brusco NK, et al. Barriers and enablers to uptake of a contemporary guideline-based management program for hip and knee osteoarthritis: A qualitative study. Osteoarthr Cartil Open. 2020;2(4):100095. Published 2020 Aug 26. doi:10.1016/j.ocarto.2020.100095

VERSION 1 - AUTHOR RESPONSE

Reviewer: 1
Dr. Hong Ji, Nursing department, The First Affiliated Hospital, Shandong First Medical University

Comments to the Author:
Overall, this qualitative study is well-structured and demonstrates clinical relevance.

Authors answer:

> Thank you for your constructive comments. We have answered them underneath.

However, I recommend the following revisions:

1. Study Sample: Please explain the rationale for achieving data saturation or sample size saturation in the study.

Authors answer:

> We thank the reviewer for this comment. In qualitative research, data saturation is used to determine when enough interviews have been conducted to capture the perspectives relevant to the research questions. In our study, data saturation was considered achieved when no new topics or themes emerged during the interviews. This approach ensures that the collected data were sufficient to address the study objectives while avoiding unnecessary additional interviews.

Authors action:

> We have added the following sentence to the "Data Collection" section:
> *"Data saturation was considered reached when no new topics emerged during the interviews. This approach was used to ensure that the data adequately captured a representative range of participant perspectives."*

2. Data collection: Please supplement the interview framework for the semi-structured interviews.

Authors answer:

> Thank you for this suggestion, we have now uploaded the interview guide as Supplementary File: interview guides.

Authors action:

> We have added the following sentence in section to the "Data Collection" section of the manuscript: *"The complete interview guides are available in Supplementary File 2."* Additionally, we have included three interview guides (for individuals with knee OA, GPs and lifestyle coaches) as Supplementary File: interview guides.

3. The research themes and subthemes would be more clearly presented if illustrated with logical diagrams.

Authors answer:

We thank the reviewer for this helpful suggestion. We agree that a visual representation could improve clarity of the results.

Authors action:

We added a figure illustrating the four CFIR domains and the corresponding constructs identified in the study. This complements the tables.

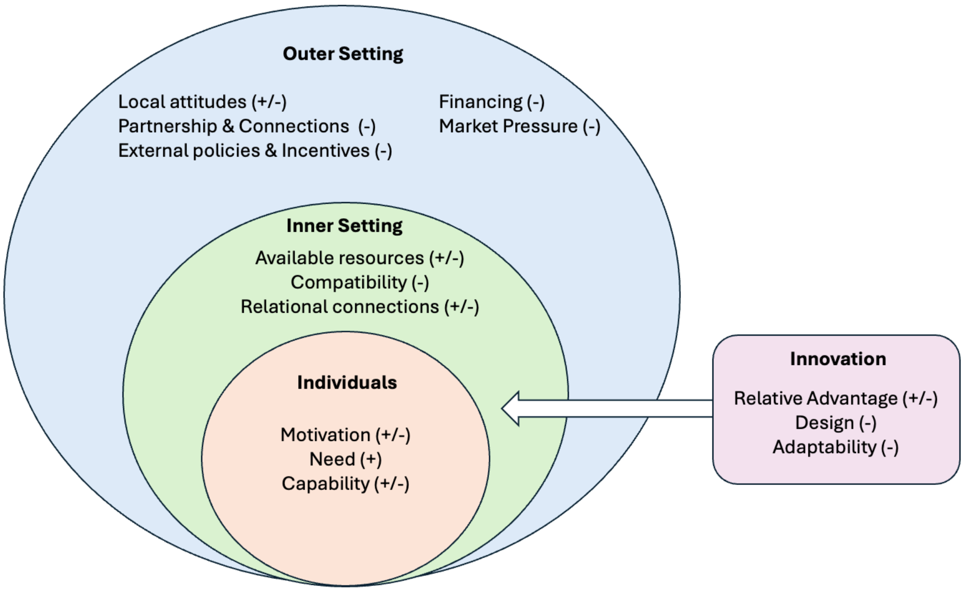

Figure 1. Identified CFIR constructs and their impact on the implementation of the CLI for individuals with knee OA and overweight. '–' indicated barriers; '+' indicated facilitators; CFIR, Consolidated Framework for Implementation Research; CLI, Combined Lifestyle Intervention; OA, osteoarthritis.

4. Please provide details on whether ethical approval was obtained prior to the study.

Authors answer:

The Medical Ethics Review Board has assessed this study and concluded that it does not fall under the scope of the Dutch Medical Research Involving Human Subjects Act (WMO). Therefore, ethical approval was not required. We will revise the sentence in the Methods section accordingly.

Authors action:

We revised the sentence in the method section: *"The Medical Ethics Review Board of Erasmus MC has assessed the study and concluded that this study did not fall under the Medical Research Involving Human Subjects Act (WMO) (MEC-2023-0756).''*

Reviewer: 2
Ms. Natalie Pavlovic, South Western Sydney Local Health District Fairfield Hospital Library, University of New South Wales South Western Sydney Clinical School

Comments to the Author:
Reviewer comments
Thank you for an interesting qualitative study exploring the factors influencing the implementation of a combined lifestyle intervention for patients with knee osteoarthritis and overweight. I have some comments for consideration.

Authors answer:

Thank you for the opportunity to revise the paper and for your constructive feedback.

ABSTRACT

1. Under the 'design' subheading please state that this is a qualitative study.

Authors answer:

Thank you for this suggestion, we added that this is a qualitative study.

Authors action:

We revised the sentence in the design section "Semi-structured interviews were conducted in a qualitative study with 23 individuals with knee OA and overweight and 16 HCPs (general practitioners (GPs) and lifestyle coaches)."

2. Under the 'strengths and limitations' section, the abbreviation LITE is used for the first time (page 3 of the manuscript). Please type out the abbreviation in this section.

Authors answer:

We thank the reviewer for pointing this out. The abbreviation LITE has now been spelled out in the '*strengths and limitations' section*

Authors action:

We have revised the text: “*By including individuals with knee OA and HCPs from various CLI programs outside the Lifestyle Intervention Trial (LITE study) and using purposive sampling, we ensured a diverse sample in terms of age, sex, socioeconomic status, health literacy, cultural background, and session attendance.”*

3. Under the ‘what is already known on this topic’ section it is stated that “the implementation of a Combined Lifestyle Intervention for this specific patient group in primary care remains poorly understood”. However, there are existing qualitative studies exploring the barriers and enables to such programs for this patient group. For example:

• Lawford BJ, Bennell KL, Jones SE, Keating C, Brown C, Hinman RS. "It's the single best thing I've done in the last 10 years": a qualitative study exploring patient and dietitian experiences with, and perceptions of, a multi-component dietary weight loss program for knee osteoarthritis. Osteoarthritis Cartilage. 2021 Apr;29(4):507-517. doi: 10.1016/j.joca.2021.01.001. Epub 2021 Jan 9. PMID: 33434629.

• Barriers and enablers to the implementation of the Australian Osteoarthritis Chronic Care Program (OACCP). Eyles, J.P. et al. Osteoarthritis and Cartilage, Volume 28, S446.

• Wallis JA, Ackerman IN, Brusco NK, et al. Barriers and enablers to uptake of a contemporary guideline-based management program for hip and knee osteoarthritis: A qualitative study. Osteoarthr Cartil Open. 2020;2(4):100095. Published 2020 Aug 26. doi:10.1016/j.ocarto.2020.100095

Authors answer:

We thank the reviewer for mentioning these relevant studies. We acknowledge that qualitative research has explored barriers and facilitators to lifestyle interventions for patients with knee OA. However, our study provides a unique contribution by focusing specifically on the implementation of CLIs in Dutch primary care, capturing perspectives of both patients and multiple types of healthcare professionals.

Authors action:

We revised the “What is already known on this topic” section to acknowledge existing qualitative studies:

“Weight loss achieved through exercise therapy and dietary changes is effective in managing knee OA symptoms and improving quality of life. Previous qualitative

studies have explored barriers and facilitators to lifestyle interventions for patients with knee OA. However, the implementation of CLIs in Dutch primary care for this specific patient group remains poorly understood."

METHODS

1. Can a brief description of the combined lifestyle intervention be provided as part of an appendix / supplementary file? The appendix could also acknowledge all of the combined lifestyle interventions that participants were recruited from (if that information is available).

Authors answer:

We thank the reviewer for this suggestion. We have included an overview of the CLI programs as a supplementary file.

Authors action:

We have created Supplementary File Table S1, which includes an overview table of the CLI programs that participants were recruited from, including specific target group, primary healthcare provider, key features, physical activity sessions, transition to regular exercise programs, and delivery format. We added the sentence: "An overview of the CLI programs is provided in Supplementary File Table S1." To the first part of the methods.

2. Under the 'study design and conceptual framework' section please add a sentence stating that the data are reported using the Standards for Reporting Qualitative Research (SRQR) criteria and include a reference for this criteria.

Authors answer:

We thank the reviewer for this suggestion. We have added a sentence to the "study design and conceptual framework" stating that the data are reported using the Standards for Reporting Qualitative Research (SRQR) criteria.

Authors action:

We added the following sentence to the manuscript:
"Data are reported following the Standards for Reporting Qualitative Research (SRQR) criteria [19]."

3. Please add a sentence in the manuscript on whether there were any exclusion criteria when recruiting participants for this study.

Authors answer:

> Thank you for the comment. No exclusion criteria were applied beyond the inclusion criteria; participants were required to be currently enrolled in or have previously participated (e.g. complete attendance or early program disengagement) in a CLI program and have knee OA.

Authors action:

> We added the following sentence to "Study sample":
> *"Participants were required to be currently enrolled in or have previously participated in a CLI program and have knee OA; no other exclusion criteria were applied."*

4. Please comment in the manuscript on the background of the interviewers to ensure transparency. For example, are they experienced qualitative researchers? Do they have a specific clinical background?

Authors answer:

> We have added information on the interviewers' backgrounds to ensure transparency. While PG and NJ had limited prior experience in qualitative research, both received training in qualitative interviewing before data collection.

Authors action:

> Added the following sentence to the "Data Collection" section: *"PG has a background in health sciences, and NJ has a background in human movement sciences. Both researchers received training in qualitative interviewing prior to the study."*

5. Please include your interview guide either as part of your manuscript (e.g. as a table or figure) or the appendix for transparency.

Authors answer:

> Thank you for this suggestion, we have now uploaded the interview guide as Supplementary File: Interview guides.

Authors action:

We have added the following sentence in section to the “Data Collection” section of the manuscript: *“The complete interview guides are available in Supplementary File 2.”* Additionally, we have included three interview guides (for individuals with knee OA, GPs and lifestyle coaches) as Supplementary File: interview guides.

6. Please add a sentence in the manuscript on whether pilot interviews were conducted to test the interview guide.

Authors answer:

We appreciate the reviewer’s comment. The initial version of the interview guide was pilot tested internally among the research team with one healthcare professional and refined based on feedback before being used in the study. We have added this information to the “Data Collection” section.

Authors action:

We have revised the “Data Collection” section:
“Three tailored semi-structured interview guides were designed, based on the CFIR domains and existing literature, for individuals with knee OA, GPs, and lifestyle coaches. These included questions based on the CFIR domains, along with additional topics relevant to the study and informed by existing literature. The initial version was pilot tested with a physiotherapist on the research team to assess clarity and relevance. Minor revisions were made to the guide after the first interview, primarily to refine the wording of questions. The complete interview guides are available in Supplementary File: interview guides 2.”

7. Please add a sentence in the manuscript on whether any repeat interviews were undertaken.

Authors answer:

We thank the reviewer for this comment. No repeat interviews were undertaken in this study.

Authors action:

We have added the sentence “*No repeat interviews were conducted.”* To the data collection section.

8. Please add a sentence in the manuscript on whether transcripts were returned to participants for comment and/or correction (i.e. member checking).

Authors answer:

We thank the reviewer for this comment. Transcripts were not returned to participants for comment or correction, none of the participants wanted to check their transcript.

Authors action:

We added to the data collection: "and none of the participants wanted to check the transcripts."

9. Under the 'data analysis' section, the author describes the coding process then states, "descriptive summaries were created for each theme to draw conclusions" (page 9). Please elaborate how the research team developed themes from the codes. For example, were themes formed by linking related codes then mapped onto components/constructs of the CIFR domains?

Authors answer:

We thank the reviewer for this comment. We have now clarified how themes were developed from codes. After initial coding, related codes were grouped together to form preliminary themes. These preliminary themes were then discussed within the research team to ensure consensus and consistency. Subsequently, the themes were mapped onto the relevant constructs of the CFIR domains to organize the data in relation to the conceptual framework.

Authors action:

We have revised the "Data Analysis" section:
"First, two researchers (PG, NJ) independently coded two transcripts using an inductive approach to develop initial codes. These codes were then organized into two separate coding trees—one for people with knee OA and one for HCPs—to account for the different perspectives and experiences between the two groups. The coding trees were subsequently refined based on discussion with a third researcher (DS) and insights from the initial transcripts, after which a third transcript was coded using the revised coding tree. After reaching 75% interrater reliability of the coding trees, one researcher (PG) coded all remaining transcripts.

Related codes were grouped to form preliminary themes, which were discussed by multiple members of the research team (PG, NJ, MvM, DS) to resolve discrepancies. The themes were subsequently mapped onto the constructs of

the four CFIR domains used in this study. Descriptive summaries were created for each theme to draw conclusions".

10. The methods sections should include a brief paragraph explaining how the researchers ensured the rigour of the findings (credibility, transferability, dependability and confirmability).

Authors answer:

We thank the reviewer for this important comment. We have added a paragraph to the method section describing how rigor was ensured.

Authors action:

We have added this paragraph to the method section: *"To ensure the rigor of the findings, several strategies were used. Credibility was enhanced through data triangulation across participant groups. Dependability was ensured using a structured coding process, including interrater reliability checks and team discussions to resolve discrepancies. Confirmability was promoted by mapping the final themes onto the constructs of the CFIR framework, supporting transparency in the analysis process."*

RESULTS

1. Please comment in your manuscript whether anyone approached for interview declined to participate.

Authors answer:

We thank the reviewer for this comment. There were several individuals (people with knee OA and GPs) who were approached and declined to participate due to other health conditions and time constraints.

Authors Action:

We have added the following sentence to the first paragraph of the results: *"In addition, several other individuals who were approached for participation declined due to other health conditions or time constraints."*

2. In table 1, the socioeconomic status is determined by the participants' highest level of education obtained. However, there may be participants with lower levels of education who have higher socioeconomic status. It may be more appropriate to list the

highest level of education attained by your participants in the table instead.

Authors answer:

> We thank the reviewer for this comment. We acknowledge that socioeconomic position (SEP) is a complex construct, and that educational level alone may not fully capture SEP. We have changed the SEP to the highest education instead. An we will acknowledge the limitation of the proxy for SEP in the discussion.

Authors Action:

> We changed SEP in Table 1 in to level of education. And we changed all 'socioeconomic status' named in the text to level of education. We also added in the "Strengths and Limitations" section a sentence to acknowledge the use of education as a proxy for SEP. "*In this study, we used educational level. This could be used as a proxy for socioeconomic position, acknowledging that this measure does not encompass all dimensions of socioeconomic position.*"

3. Adding to the above point, in the methods, it is stated that employment status was collected. Can this information be included in table 1 as well? For example, employed, unemployed, retired etc.

Authors answer: Thank you for this comment. We don't have for all the participants the employment status. We therefore decided to delete the employment status data collection from the method section, to avoid the confusion.

Authors action: We deleted the "employment status" in the data collection.

4. In table 1, 'Caucasian', 'Asian' and 'Mixed/multiple' are listed as ethnicities. However, these are not ethnicities. Examples of ethnic groups include Dutch, English, French, Chinese, Vietnamese, Assyrian etc. Please include the participants' ethnicities in the table.

Authors answer:

> We thank the reviewer for this comment. Detailed ethnic information was available for our participants. Four participants identified as Surinamese, 14 as Dutch, and five as of mixed Dutch/other ethnic background. One HCP identified as non-western (not specified), 14 as Dutch, and one as of mixed Dutch/other ethnic background.

Authors action:

We have revised Table 1 to list participants' ethnicities as follows: Dutch (n = 14), Surinamese (n = 4), and mixed Dutch/other (n = 5). HCP: Dutch (n=14), Non-western (not specified) (n=1), and mixed Dutch/other (n=1)

5. In table 1, the healthcare professionals' occupations are listed as 'Lifestyle Coach/Dietitian' (n=8) and 'Exercise professional' (n=1). Later in the text it is stated that nine lifestyle coaches were interviewed. Can you please update your table to accurately reflect this? For example, it can be recorded as lifestyle coach (dietitian) and lifestyle coach (exercise professional).

Authors answer:

We thank the reviewer for pointing out this inconsistency. We have revised Table 1 to distinguish between lifestyle coaches with different professional qualifications ensuring consistency with the text.

Authors Action:

We updated Table 1 as follows:

- Lifestyle coach (dietitian) n = 8
- Lifestyle coach (exercise professional) n = 1

6. In table 1 can you also clarify what is meant by 'exercise professional'? For example, is it a physiotherapist, exercise physiologist or personal trainer?

Authors answer:

Thank you for pointing this out. The "exercise professional" in Table 1 refers to a personal trainer involved in delivering the BeweegKuur program.

Authors action:

We added a note to the table description to specify that the exercise professional was a personal trainer. We also clarified it in the text:
"The exercise professional was a qualified personal trainer who specifically delivered the BeweegKuur program."

7. Do you have data on the mean BMI and range in the patient group? If so, can it be included in Table 1?

Authors answer:

We thank the reviewer for this comment. BMI data were not collected for all participants. Therefore, we are unable to provide mean or range BMI. They all have a BMI higher than 25 to be eligible for CLI.

Authors Action:

No changes were made to Table 1. But we added in the text in the first sentence of the results, that all participants had BMI > 25.

8. Can an additional table be included outlining the characteristics for each individual patient participant? It can include their sex, age, BMI, education level, employment status, ethnicity/cultural background, attendance rate. This will provided context to the quotes provided by participants in your results section.

Authors answer: We do not have all the information of the participants that you requested but we have added a table with characteristics. We do not have the BMI and the employment status of the participants.

Authors action: We have added a table with the characteristics in the participants in the Supplementary files. And we added this sentence in the Result section: "For detailed information about the participants, see Supplementary file Table S2."

9. Please comment on whether the participants provided feedback on the findings (i.e. member checking).

Authors answer:

We thank the reviewer for this comment. Participants were not asked to provide feedback on the findings. We have had focus groups to discuss the results and think about strategies how to improve the CLI for people with knee OA and overweight. But this is outside the scope of this manuscript and will be a topic to discuss in the follow-up paper.

Authors Action:

No changes were made to the manuscript.

10. Complexity is listed as one of the themes in your study. However, the quotes provided by the participants in Table 3 for 'complexity' reference the duration of the program which is more related to the 'design' theme. Can you please provide quotes that are more relevant to the 'complexity' theme?

Authors answer:

Thank you for your thoughtful feedback. We agree that there was overlap between some of the themes under the Innovation domain. Based on your suggestion, we combined the themes 'design' and 'complexity' into a single theme "Design".

Authors action:

We updated the Results section to reflect the combined theme "Design," merging the previous 'design' and 'complexity' themes. Corresponding text, including the in-text quote references, as well as tables 2 and 3 have been revised.

11. I notice there are similarities between the themes listed. For example, under the innovation domain, the 'design', 'adaptability' and 'complexity' themes are all related to the design of the CLI program. Can these be grouped as one theme?

If the themes must remain separate and named according to the constructs in the CIFR, the data analysis section in the methods needs to describe this approach (see my comment under the methods section).

Authors answer:

Thank you for your feedback. We agree that there was overlap between some of the themes under the Innovation domain. Based on your suggestion, we combined the themes 'design' and 'complexity' into a single theme (see previous action). "Adaptability'' was kept as a separate theme to reflect how participants experienced the ability of the CLI to be personalized or adjusted to their specific needs and contexts, consistent with the CFIR framework.

Authors action:

We updated the Results section to reflect the combined theme "Design," merging the previous 'design' and 'complexity' themes. The theme 'adaptability' remains separate under the Innovation domain.

12. As I continue to read the manuscript, I can see themes in the text are named differently to the CFIR themes listed in Table 2.

a) Based on what I have read your themes are as follows:

Innovation domain
Theme:
• Perceived effectiveness of the CLI for OA Management

• Insufficient physical activity support and program limitations in the CLI for individuals with knee OA
• Challenges in participation due to diverse knowledge levels and health literacy

Outer setting domain
Theme:
• Sociocultural factors and social support
• Scepticism among GPs regarding the implementation of the CLI

Inner setting domain
Theme:
• Fit to coach

Individuals domain
Theme:
• Participant motivation and engagement in lifestyle change
• Expertise among lifestyle coaches for OA support

Author answer: This is correct. We have adapted Table 2 and 3 according to your valuable comments to make it more clear.

b) Table 3 should be amended so that the themes in the manuscript are listed under the CFIR domains. The participant quotes should correspond to your themes in the table.
Authors answer: Thank you for this constructive feedback. We have adapted Table 3 and added the Themes that we describe in the results.

Authors action: See adaptation of Table 3

c) Table 2 should then be amended. Instead of 'CFIR theme' it should read 'CFIR construct'. Instead of 'definition of theme' it should be 'definition of construct'. If appropriate, you may also add an additional column (e.g. named 'corresponding theme') to list the themes that are mapped to that domain / construct.

Authors answer:

> We thank the reviewer for this suggestion. We have amended Table 2 to replace the column headings 'CFIR Theme' and 'Definition of theme' with 'CFIR Construct' and 'Definition of construct'.

Authors action:

Table 2 has been revised as follows:

- Column headings changed from 'CFIR Theme' to 'CFIR Construct' and 'Definition of theme' to 'Definition of construct.' And we have added a column with corresponding theme.

DISCUSSION

1. Under the 'strengths and limitations' section it is stated that this is the first qualitative study examining the implementation of the CLI for patients with knee OA from both participant and HCP perspectives. However, there are existing qualitative studies exploring the barriers and enables to such programs from patients and HCPs. For example:

• Lawford BJ, Bennell KL, Jones SE, Keating C, Brown C, Hinman RS. "It's the single best thing I've done in the last 10 years": a qualitative study exploring patient and dietitian experiences with, and perceptions of, a multi-component dietary weight loss program for knee osteoarthritis. Osteoarthritis Cartilage. 2021 Apr;29(4):507-517. doi: 10.1016/j.joca.2021.01.001. Epub 2021 Jan 9. PMID: 33434629.

• Barriers and enablers to the implementation of the Australian Osteoarthritis Chronic Care Program (OACCP). Eyles, J.P. et al. Osteoarthritis and Cartilage, Volume 28, S446.

• Wallis JA, Ackerman IN, Brusco NK, et al. Barriers and enablers to uptake of a contemporary guideline-based management program for hip and knee osteoarthritis: A qualitative study. Osteoarthr Cartil Open. 2020;2(4):100095. Published 2020 Aug 26. doi:10.1016/j.ocarto.2020.100095

Authors answer:

We thank the reviewer for highlighting these relevant studies. We acknowledge that previous qualitative research has explored barriers and facilitators to lifestyle interventions for patients with knee OA from the perspectives of both patients and healthcare professionals. To clarify, our study specifically examines the implementation of CLIs in Dutch primary care, incorporating the perspectives of both individuals with knee OA and multiple types of healthcare professionals'.

Authors action:

We revised the first sentence of the "Strengths and limitations" section to clarify the focus of our study: "*Earlier studies have explored barriers and enablers for*

lifestyle programs, but to our knowledge, this is the first qualitative study examining the implementation of the CLI in Dutch primary care for patients with knee OA from both participant and HCP perspectives."
